# Effects of Compound Chinese Herbal Medicine Additive on Growth Performance and Gut Microbiota Diversity of Zi Goose

**DOI:** 10.3390/ani12212942

**Published:** 2022-10-26

**Authors:** Jinlei Zheng, Shuang Liang, Yan Zhang, Xueqi Sun, Yumei Li, Jizhe Diao, Liping Dong, Hongyu Ni, Yijing Yin, Jing Ren, Yuwei Yang, Yonghong Zhang

**Affiliations:** 1 College of Animal Science, Jilin University, Changchun 130062, China; 2 College of Animal Science and Technology, Jilin Agriculture Science and Technology University, Jilin 132109, China; 3 Jilin Academy of Agricultural Sciences, Changchun 130062, China

**Keywords:** Compound Chinese Herbal Medicine Additive, growth performance, slaughter performance, gut microbiota, Zi goose

## Abstract

**Simple Summary:**

Responsible use of antibiotics is essential to the health of animals and humans. Chinese herbs are good alternative to antibiotics, with safe ingredients and no drug synthetic compound residues in tissues. Compared with the use of single herbs, the rational combination of multiple herbs can produce synergistic effects, while achieving improved economic benefits. The specific Compound Chinese Herbal Medicine Additive (CCHMA) used in this study consisted of *Astragalus*, *Licorice*, *Radix Codonopsis*, *Citri Reticulatae Pericarpium*, *Angelica Sinensis*, *Atractylodis* and *Cimicfugae* *Rhizoma* in the ratio of 6:6:4:3:3:1:1. The results showed that CCHMA had no adverse effects on Zi goose. At the same time, CCHMA improved the performance parameters of Zi goose and eliminated the risk of antibiotic residues in meat.

**Abstract:**

This study investigated the effects of CCHMA on growth performance, slaughter performance, serum biochemical indicators, intestinal morphology and microbiota of Zi goose. Initially, it was determined the optimal addition concentration of CCHMA to be 3 g/kg by the first feeding experiment. Then, 78 Zi geese were divided into control and CCHMA supplemented groups. The results showed that the body weight (BW) and average daily gain (ADG) of the CCHMA supplemented group was significantly increased (*p* < 0.05), and the feed/gain (F/G) of the CCHMA supplemented group was significantly decreased (*p* < 0.05) compared with the control group. The dressed yield percentage in the CCHMA supplemented group significantly increased by 0.78% (*p* < 0.05). Alanine aminotransferase (ALT) and aspartate aminotransferase (AST) levels were significantly lower in the CCHMA fed birds than in the control group (*p* < 0.05). Further, 16S rDNA gene sequencing conducted for cecal flora composition found that 3 g/kg CCHMA significantly increased the abundance of beneficial bacteria (*CHKCI001, Colidextribacter and Subdoligranulum*) (*p* < 0.05; *p* < 0.01) and suppressing harmful bacteria (*Bacteroidetes and Methanobrevibacter*) (*p* < 0.05) in the cecum of Zi goose. In conclusion, adding 3 g/kg of CCHMA in the diet can improve the growth performance, slaughter performance of Zi goose, and optimize the cecum microflora.

## 1. Introduction

The use of antibiotics can produce drug residues in tissues, antimicrobial resistance and other problems [1]. Therefore, the European Commission and the United States Food and Drug Administration announced bans on the use of antibiotics as growth enhancers in animal feed in 2005 and 2017, respectively [2].

Alternatively, herbs are natural medicinal plants, and the plant extracts produce no drug synthetic compound residues in tissues and exhibit anti-bacterial and anti-inflammatory properties [3,4,5], which have proved to be effective in the search for alternatives to antibiotics [6,7]. The specific Compound Chinese Herbal Medicine Additive (CCHMA) used in this study consisted of *Astragalus*, *Licorice*, *Radix Codonopsis*, *Citri Reticulatae Pericarpium*, *Angelica Sinensis*, *Atractylodis* and *Cimicfugae Rhizoma* in the ratio of 6:6:4:3:3:1:1. Many reports have pointed out that adding herbs such as *Astragalus* and *Licorice* can improve the growth performance of piglets and quail [8,9]. *Astragalus* contains a variety of active ingredients, such as flavonoids, saponins and polysaccharides [10]. Feeding of broiler chickens with *Licorice* and *Citri Reticulatae Pericarpium* can have antioxidant effects, mainly due to the antioxidant and anti-inflammatory effects of triterpenoid saponins and phenolic compounds in *Licorice* and flavanone glycosides and polymethoxyflavones in *Citri Reticulatae Pericarpium* extract [11]. Compared with the use of single herbs, CCHMA can significantly improve efficacy and produce synergistic effects. After CCHMA enters the gastrointestinal tract, flavonoids and saponins are broken down into glycosides by the action of intestinal flora enzymes and exert their pharmacological activities.

Chinese herbs could enhance intestinal mucosal immune function [12]. A recent study has found that the supplementation of the *Astragalus* polysaccharides improved intestinal morphology of ducklings [13]. The cecum is the largest major reservoir of bacteria in poultry. And this is especially apparent for the geese, which have a more developed paired cecum compared to other poultry [14]. The rich microbiota in the cecum will help the geese to better ferment the indigestible carbohydrates. Gut microbiota is an important indicator of animal health. It was found that Chinese herb medicine mixture could up-regulate the abundance of beneficial flora in the broiler intestine (*Lactobacillus* and *Candida*), enhance intestinal immune mechanisms and accelerate intestinal motility [15,16]. In this experiment, CCHMA was added to the feed in a reasonable combination of seven herbs to improve the structure of the cecum flora, thereby improving the quality of animal products and ultimately achieving higher economic benefits.

Chinese geese industry now occupies a dominant position in the world poultry industry [17]. The Zi goose selected for this experiment is one of the excellent local breeds in China, distributed only in the northeast of China, with the advantages of cold resistance, roughage-resistance, good meat quality and high egg production [18]. However, there are few studies on CCHMA for meat goose. The aims of this study were to investigate the effects of CCHMA on the growth performance, slaughter performance, serum biochemical indicators, intestinal morphology and gut microbiota of Zi goose.

## 2. Materials and Methods

### 2.1. Animals, Diet and Experimental Design

A total of 114 Zi geese were involved in this research of two feeding experiments. Half with the number of female and half with the number of male Zi geese. All the Zi geese were provided by Jilin Province Jiuzhou Fei Goose Animal Husbandry Technology Co., Ltd. (Baicheng, China). All experimental protocols were approved by the College of Animal Science of Jilin University Ethics Committee (SY202105020). The specific CCHMA was provided by Jilin Kangfa Antibiotics-free Ecological Agriculture and Animal Husbandry Technology Development Co. (Changchun, China), and the composition was *Astragalus*, *Licorice*, *Radix Codonopsis*, *Citri Reticulatae Pericarpium*, *Angelica Sinensis*, *Atractylodis* and *Cimicfugae* *Rhizoma* in the ratio of 6:6:4:3:3:1:1.

In the first feeding experiment, to determine the optimal concentration of CCHMA, 36 adult Zi geese with no significant difference in body weight divided into 4 groups of 9 animals each (*n* =  3 biological replicates per group). The 4 groups were the control group (CON), 2 g/kg of CCHMA in feed, 3 g/kg of CCHMA in feed, and 4 g/kg of CCHMA in feed. All geese in outdoor terrestrial captivity, with free feeding and watering, natural light and ventilation, manual feeding and routine immunization. The experiment lasted for 70 days. The optimal concentration of CCHMA was determined to be 3 g/kg (experimental data are shown in the Results).

In the second feeding experiment, a total of 78 one-day-old Zi geese were allotted to 2 groups randomly (each group consisted of 3 replications with 13 geese per replicate). Half with the number of female and half with the number of male Zi geese. The control group was fed a basal diet and 3 g/kg of CCHMA was added to the basal diet in the treatment group. The goose house was thoroughly cleaned and disinfected to reduce bacterial contamination before the geese moved in. The first 4 weeks were the brooding period and all Zi geese lived indoors. The temperature in the house was controlled at 30–31 °C for the first week and then decreased 2–3 °C per week until the geese adapted to outdoor temperature (25–30 °C) in the fifth week and the ambient humidity was controlled at 60–65%. At the end of brood rearing, all Zi geese were kept in outdoor terrestrial captivity. Indoor light and natural light and ventilation, free feeding and watering, and routine immunization were provided according to standard protocol. The experiment also lasted for 70 days. The diet formulations and nutritional tables are shown in Table 1.

### 2.2. Sample Collection

From each group at 70 days of age, five geese whose weight was close to the average weight of the group were randomly selected and 5 mL of blood was collected from each goose wing vein and collected in a centrifuge tube. Then, within each group, ten Zi geese were weighed and sacrificed by exsanguination. Samples of the ileum (13 cm before the ileocecal junction) with a length of about 5 cm were collected, rinsed with saline and soaked in 10% formalin solution for 72 h. The contents of the mid-section of each Zi goose cecum were collected in frozen storage tubes using aseptic technique, then quickly placed on dry ice and sealed, and these samples were immediately sent out for testing. After slaughtering, the carcass weight, half evisceration weight, full evisceration weight, breast muscle weight, leg muscle weight, and abdominal fat weight were measured.

### 2.3. Performance Parameter Measurements

The initial weight and final weight of a total of 114 Zi geese was recorded separately during the experiment, the feed intake was measured once a week, and average daily gain (ADG) (g), average daily feed intake (ADFI) (g), feed/gain (F/G) were calculated for each group of Zi goose on day 70.

### 2.4. Slaughter Performance

Slaughter indicators including dressed yield (%), half-eviscerated yield (%), all-eviscerated yield (%), breast muscle yield (%), leg muscle yield (%) and abdominal fat yield (%) were calculated using standard methods [19].

### 2.5. Serum Biochemical Indicators

The collected blood was allowed to stand at room temperature until it was completely coagulated and centrifuged at 4000 r/min for 15 min at 4 °C. Then the serum was pipetted into 1.5 mL centrifuge tubes with a micropipette and stored at −20 °C. Biochemical indicators were measured in serum within 24 h. Serum biochemical indicators were measured using commercial (Meikang Biotechnology, Ningbo, China.) kits and an automatic biochemical analyzer (MS-880B, Meikang, Ningbo, China.). According to the manufacturer’s instructions, the following 7 serum biochemical parameters were measured: total protein (g/L), albumin (g/L), globulin (g/L), alanine aminotransferase (ALT) (U/L), aspartate aminotransferase (AST) (U/L), creatinine (mg/dL) and blood urea nitrogen (mg/dL).

### 2.6. Ileal Intestinal Morphology

Samples were embedded in paraffin wax, sectioned at 7 μm, and stained with haematoxylin and eosin (Changchun Xavier Biotechnology Co., Changchun, China). The villus height and crypt depth were measured in three replicates per goose under a light microscope (X 40 magnification) with Slide Viewer (version 2.5.0; 3DHISTECH Ltd., Budapest, Hungary)image-analyzing system and calculated the ratio of villus height to crypt depth. The measurement standards and methods were referred to Abolfathi et al. [20].

### 2.7. DNA Extraction and Cecal Microbiota Analysis

Total DNA was extracted from cecal content samples using a Magnetic Soil and Stool DNA Kit (Tiangen Biotech Co., Ltd., Beijing, China) according to the manufacturer’s protocol. DNA quantity and purity were determined by a Nanodrop 1000 spectrophotometer (Nanodrop Technologies, Wilmington, DE, USA). Amplification of V3–V4 hypervariable regions of the 16S rRNA gene was conducted with 341F CCTAYGGGRBGCASCAG and 806R GGACTACNNGGGTATCTAAT primers [21]. The amplicon libraries were sequenced using the Illumina NovaSeq platform (250-bp, pair end; Illumina, San Diego, CA, USA). Sequencing data were processed by QIIME2 (version 2019.4) software package [22]. Briefly, paired sequences were demultiplexed with “demux” plugin and then the primers were cut out with the Cutadapt plugin. Subsequently, the imported paired sequences were quality filtered (Q < 20), denoised, and merged through the DADA2 plugin to generate the amplicon sequencing variants (ASVs), and then the chimeric sequences and singleton ASVs were removed [23]. After that, nonsingleton ASVs were classified into taxa using a pretrained SILVA database (https://www.arb-silva.de/, accessed on 18 July 2022). The alpha diversity was analyzed based on the rarefied ASVs through several indices, including observed ASVs, Chao1, Shannon, and Simpson. Beta diversity was performed based on the Bray–Curtis distance to compare the overall dissimilarity of cecal bacteria between two groups samples and was presented by principal coordinate analysis (PCoA). A heatmap was generated according to major phyla (top 5 taxa with high relative abundance) and genera (top 25 taxa with high relative abundance) information using the R heatmap plugin. Species composition differences were analyzed at the phylum and genus levels to obtain the abundance differences in bacterial communities between two groups of cecal samples. The LEfSe software (Version 1.0) was used to do LEfSe analysis so as to find out the biomarkers (basic on an LDA > 3.5).

### 2.8. Statistics and Analysis

All data were analyzed using SPSS (version 23.0; SPSS Inc., Chicago, IL, USA). Data from the first feeding experiment were analyzed by one-way ANOVA with treatment and replicate as class variables. And data from the second feeding experiment were analyzed by *t*-test and Mann–Whitney test, following the general linear model’s procedure and non-parametric statistical test of SPSS with treatment and replicate as class variables, respectively. The GraphPad Prism 8 software (San Diego, CA, USA) was used to make histograms. All data are presented as least squares means ± standard deviation. The relationship between the cecal microbiota composition and the growth performance indexes (BW (g), ADG (g), F/G and ADFI (g)) were examined by using the R package (Version 2.15.3, R Foundation for Statistical Computing, Vienna, Austria) to evaluate Spearman’s rank correlation coefficient. Significant differences at *p* *<* *0.05*, 0.01 and 0.001 were indicated as *, ** and ***, respectively.

## 3. Results

### 3.1. Optimal Concentration Screening of CCHMA

**Figure 1:**
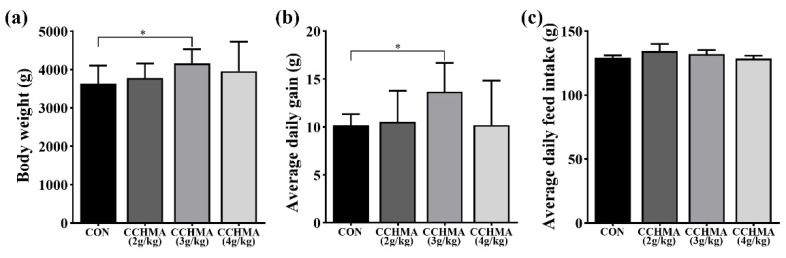
Effect of CCHMA at different concentrations on growth performance of Zi goose. (**a**) Effect of different concentrations of CCHMA on the body weight of geese. (**b**) Effect of different concentrations of CCHMA on the average daily gain of geese. (**c**) Effect of different concentrations of CCHMA on the average daily feed intake of geese. Each bar represents the mean and standard error. * *p* < 0.05 when compared with the control group.

Compared with CON, BW and ADG in the CCHMA (3 g/kg) group was significantly increased (*p* < 0.05); (Figure 1a,b). BW in the CCHMA (3 g/kg) group was higher than in the groups CCHMA (2 g/kg) and CCHMA (4 g/kg) (*p* > 0.05). However, there was no significant difference in ADFI intake on four groups; (Figure 1c). As such, it was determined the optimum amount of CCHMA to be added at 3 g/kg.

### 3.2. Growth Performance

**Figure 2:**
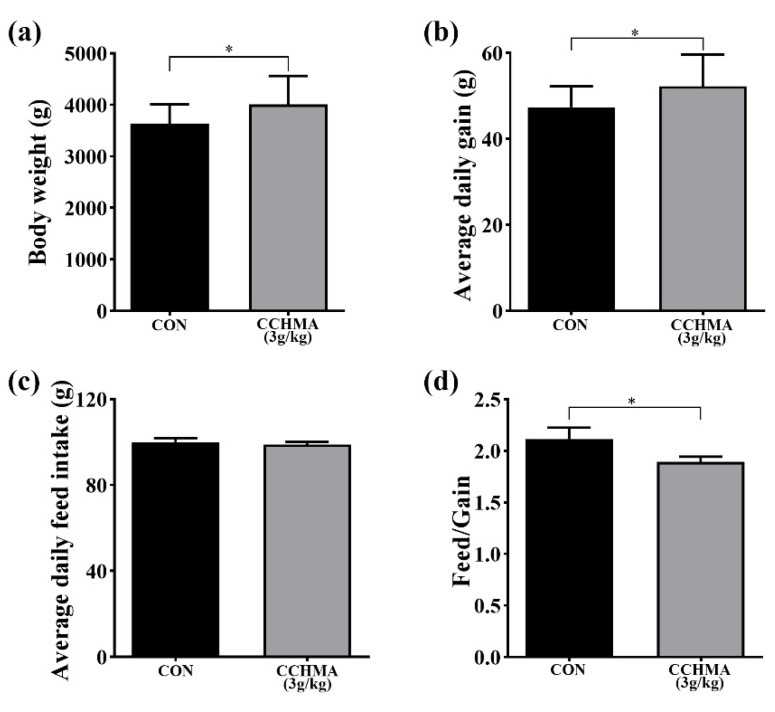
Effect of CCHMA on growth performance of Zi goose. (**a**) The body weight of geese. (**b**) The average daily gain of geese. (**c**) The average daily feed intake of geese. (**d**) The feed/gain of geese. Each bar represents the mean and standard error. * *p* < 0.05 when compared with the control group.

According to the above results, the 3 g/kg CCHMA was used for subsequent research. Compared with CON, BW and ADG of the CCHMA group were significantly higher than those of CON (*p* < 0.05); (Figure 2a,b), and there was no significant difference in feed intake among groups (*p* > 0.05); (Figure 2c). F/G of the CCHMA group was significantly lower than that of CON (*p* < 0.05); (Figure 2d).

### 3.3. Slaughter Performance

**Figure 3:**
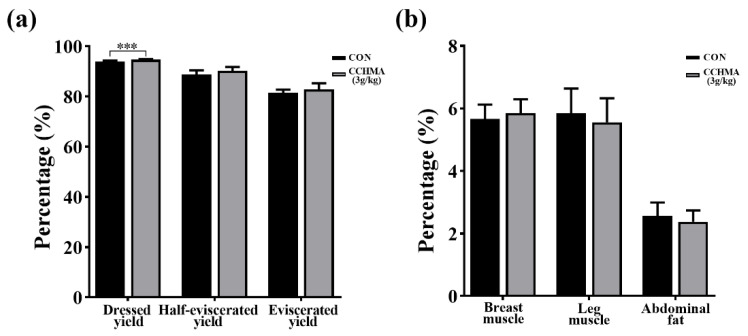
Effect of CCHMA on slaughter performance of Zi goose. (**a**) The percentage of dressed yield, half-eviscerated yield and eviscerated yield of geese. (**b**) The percentage of breast muscle, leg muscle and abdominal fat of geese. Each bar represents the mean and standard error. *** *p* < 0.001 when compared with the control group.

Compared with CON, the dressing percentage in the CCHMA group was significantly increased (*p* < 0.001). However, the other slaughter indicators, including half-eviscerated yield, eviscerated yield, breast muscle and leg muscle percentage, were not different in the CCHMA group (*p* > 0.05). And the abdominal fat yield of the CCHMA group had a downward trend (*p* > 0.05); (Figure 3).

### 3.4. Serum Biochemical Indicators

**Figure 4:**
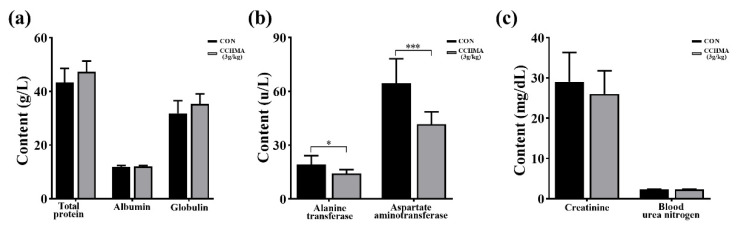
Effect of CCHMA on serum biochemical indicators of Zi goose. (**a**) The content of total protein, albumin and globulin of geese. (**b**) The content of alanine transferase and aspartate aminotransferase of geese. (**c**) The content of creatinine and blood urea nitrogen of geese. Each bar represents the mean and standard error. * *p* < 0.05, *** *p* < 0.001 when compared with the control group.

To determine if the CCHMA produced effects on the general physiology of geese, blood samples were collected for detection of serum biochemical indicators. There was no significant difference in total protein, albumin and globulin between the two groups (*p* > 0.05); (Figure 4a). Compared with CON, ALT level and AST level in the CCHMA group were significantly lower than those in CON (*p* < 0.05; *p* < 0.001); (Figure 4b). But there was no statistical difference in creatinine and blood urea nitrogen between the two groups (*p* > 0.05); (Figure 4c).

### 3.5. Ileal Intestinal Morphology

**Figure 5:**
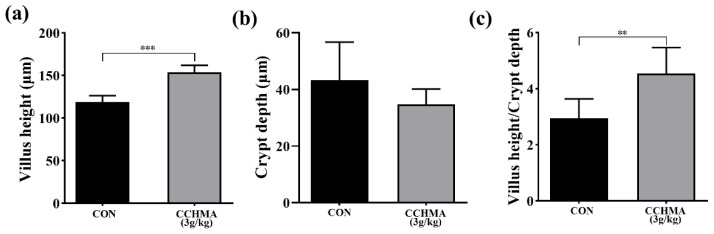
Effect of CCHMA on ileal intestinal morphology of Zi goose. (**a**) Ileal villus height in geese. (**b**) Ileal crypt depth in geese. (**c**) Ratio of ileal villus height/crypt depth in geese. Each bar represents the mean and standard error. ** *p* < 0.01, *** *p* < 0.001 when compared with the control group.

The result shown that the Zi goose ileal villus height was significantly higher in the CCHMA group (*p* < 0.001) and the ileal villus height/crypt depth was significantly higher in the CCHMA group (*p* < 0.01); (Figure 5).

### 3.6. Cecum Microbiota

Prior to assessing the efficacy of CCHMA for the treatment of Zi goose growth performance, there was performed a preliminary trial to verify the optimal concentration of CCHMA. Therefore, the following study evaluated only the effects of CCHMA (3 g/kg) on cecum microbial development of Zi goose.

#### 3.6.1. ASV Analysis of Caecum Flora

**Figure 6:**
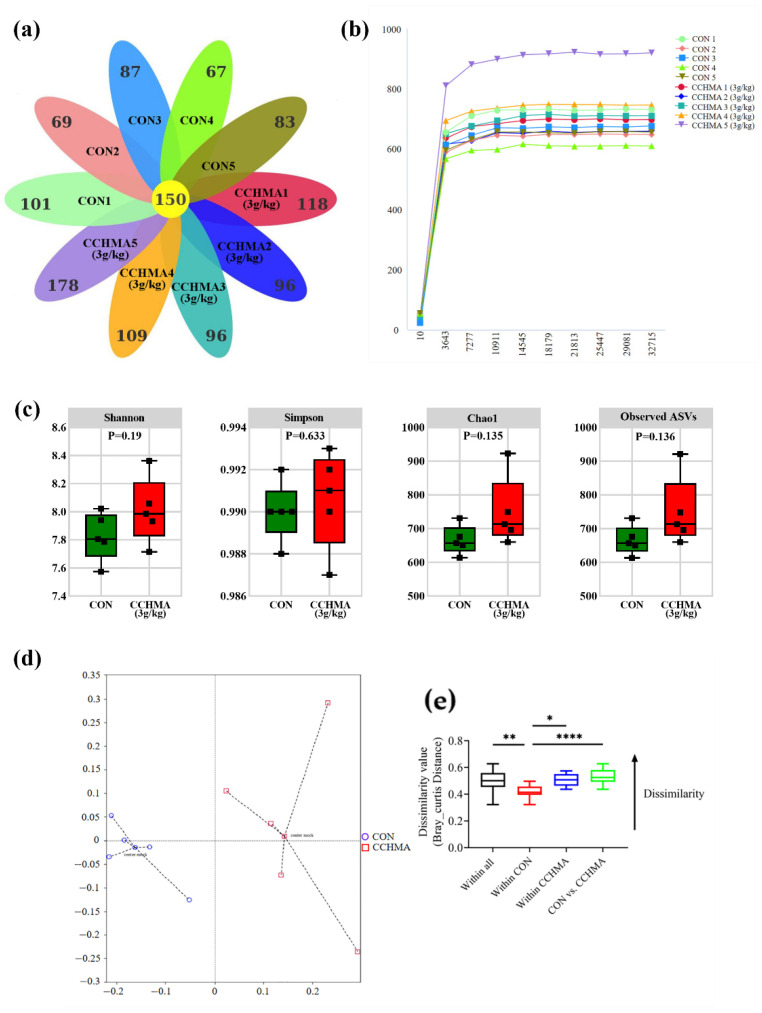
Overview of 16S rDNA gene Illumina MiSeq sequencing in cecal contents. (**a**) Petals figure. (**b**) Rarefaction curve; (**c**) Alpha diversity analysis; (**d**) PCoA two-dimensional figure based on Bray_Curtis distance analysis; (**e**) Sample distance figure based on weighted analysis. * *p* < 0.05, ** p < 0.01, **** *p* < 0.0001. when compared with the control group.

The collected cecal contents were submitted for gut microbiota analysis by sequencing the 16S rDNA genes and sequencing depths results are depicted in Supplementary Table S1. The ASVs were clustered with 100% agreement for the number of valid sequences of all samples. After high-throughput sequencing, a total of 1105 ASVs were obtained for the 10 samples, and 150 ASVs were shared among the samples (Figure 6a). The rarefaction curve was constructed through counting the Chao1 index values of the samples. The rarefaction curve was flat, indicating that the amount of sequencing data was reasonable (Figure 6b).

#### 3.6.2. Alpha and Beta Diversity Analysis

There were no significant differences in the Observed ASVs (*p =* 0.136), Chao1 (*p =* 0.135), Shannon (*p =* 0.19), and Simpson (*p =* 0.633) between the CON and the CCHMA group (Figure 6c). However, principal coordinates analysis (PCoA) based on Bray‒Curtis distance (Figure 6d,e) revealed that the bacterial communities in the cecal samples of the CON and CCHMA groups were markedly different (*p =* 0.012).

#### 3.6.3. Analysis of the Intestinal Flora Structure

**Figure 7:**
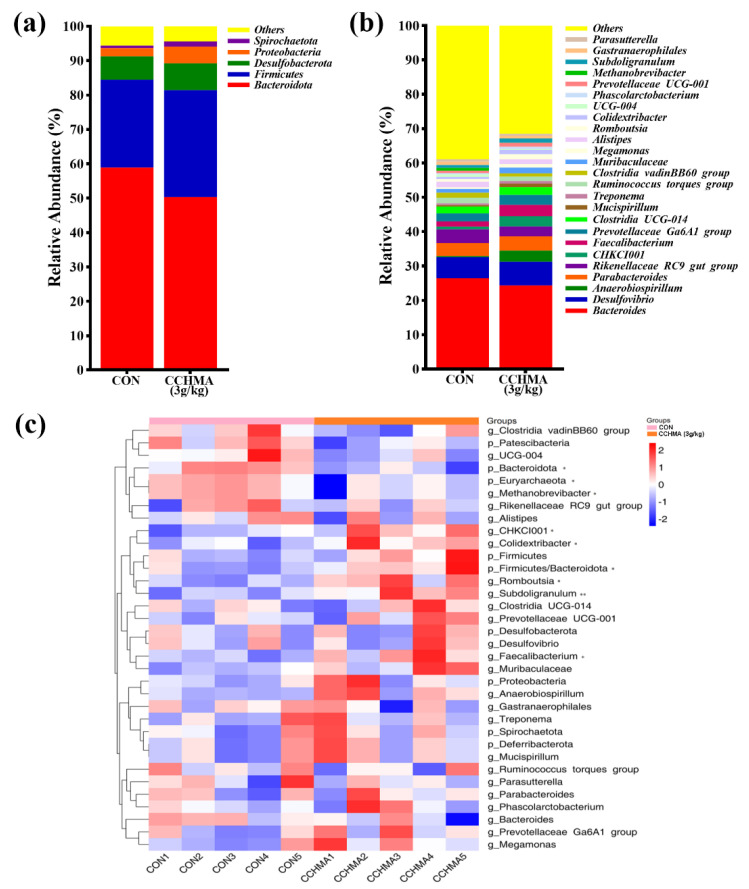
Analysis in diversity of cecal microbiota. (**a**) Histogram of species abundance at the phylum level; (**b**) Histogram of species abundance at the genus level; (**c**) Heatmap of major phyla and genera of cecal microbiota. * *p* < 0.05, ** *p* < 0.01, when compared with the control group.

The study analyzed the relative abundance of the two groups of samples at phylum and genus levels. At the phylum level, the main common bacterial phylum to the two groups were *Bacteroidota* and *Firmicutes*, accounting for more than 80% (Figure 7a). The 5 taxonomic units with the highest average abundance corresponding to the CON group and CCHMA group are *Bacteroidota* (58.91% vs. 50.30%), *Firmicutes* (25.59% vs. 31.18%)*, Desulfobacterota* (6.77% vs. 7.72%), *Proteobacteria* (2.40% vs. 4.87%) *and Spirochaetota* (0.73% vs. 1.53%); (Figure 7a).

At the genus level, *Bacteroides* and *Desulfovibrio* are the main bacterial genus in CON and the CCHMA groups (26.42% vs. 24.25%, 6.2% vs. 6.91%, respectively). And the taxonomic units with the highest average abundance corresponding to the CON group and CCHMA group are *Bacteroides* (26.42% vs. 24.35%), *Desulfovibrio* (6.20% vs. 6.91%), *Anaerobiospirillum* (0.28% vs. 3.22%), *Parabacteroides* (3.82% vs. 4.16%), *Rikenellaceae_RC9_gut_group* (3.92% vs. 2.85%), *CHKCI001* (0.83% vs. 3.00%), *Faecalibacterium* (1.50% vs. 3.28%), *Prevotellaceae_Ga6A1_group* (2.36% vs. 2.94%), *Clostridia_UCG-014* (1.95% vs. 2.24%), *Mucispirillum* (0.46% vs. 1.08%), *Treponema* (0.51% vs. 0.69%), *Ruminococcus_torques_group* (1.65% vs. 1.33%), *Clostridia_vadinBB60_group* (1.41% vs. 0.92%), *Muribaculaceae* (1.13% vs. 1.68%), *Megamonas* (0.46% vs. 1.03%), *Alistipes* (1.65% vs. 1.40%), *Romboutsia* (0.73% vs. 1.46%), *Colidextribacter* (0.64% vs. 1.23%), *UCG-004* (0.73% vs. 0.26%), *Phascolarctobacterium* (0.36% vs. 0.72%), *Prevotellaceae_UCG-001* (0.70% vs. 1.11%), *Methanobrevibacter* (0.86% vs. 0.08%), *Subdoligranulum* (0.79% vs. 1.16%), *Gastranaerophilales* (1.00% vs. 0.86%), *Parasutterella* (0.72% vs. 0.56%); (Figure 7b).

Specifically, the CCHMA group had a significantly higher abundance of *Firmicutes/Bacteroidaeota* ratio (*p =* 0.032), *Euryarchaeota* (*p =* 0.032), *CHKCI001* (*p =* 0.032), *Faecalibacterium* (*p =* 0.032), *Colidextribacter* (*p =* 0.016), *Romboutsia* (*p =* 0.032), *Subdoligranulum* (*p =* 0.008), and a lower abundance of *Bacteroidota* (*p =* 0.005) and *Methanobrevibacter* (*p =* 0.032) than CON (Figure 7c).

#### 3.6.4. Linear Discriminant Analysis Effect Size (LEfSe) Analysis

**Figure 8:**
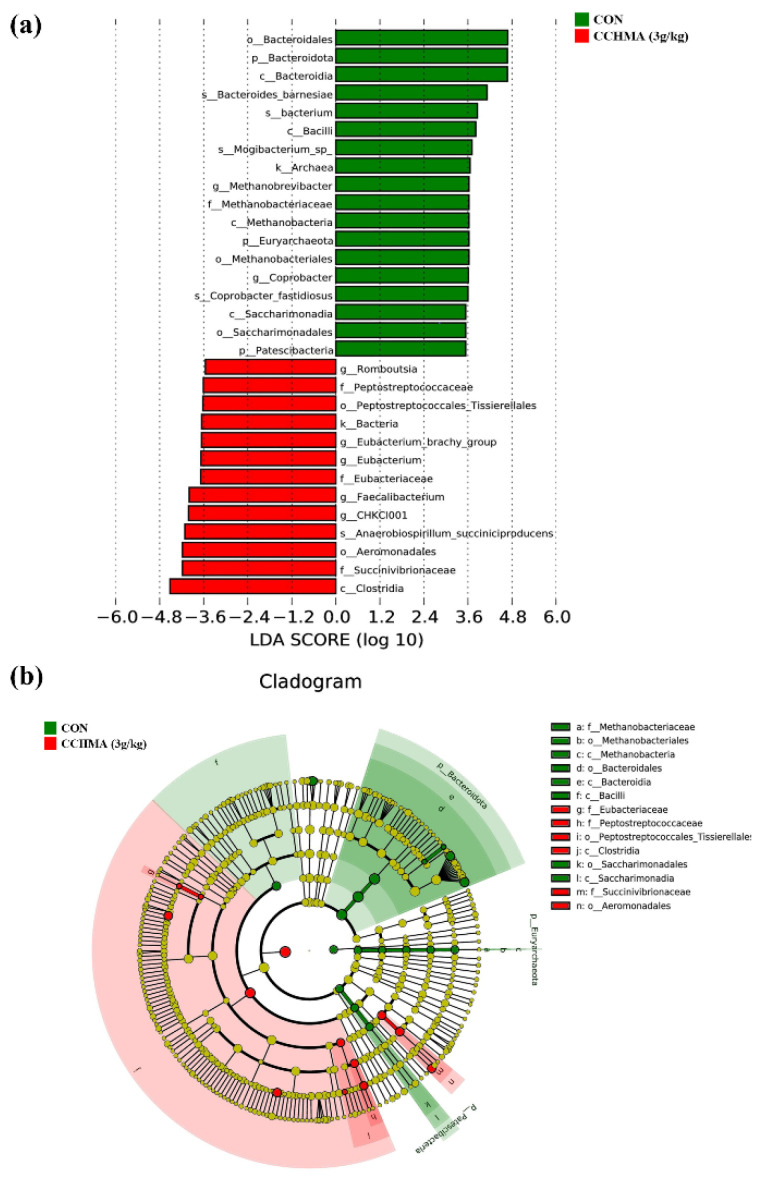
Distribution histogram, and evolutionary branch graph. (**a**) The histogram of the LDA score. The influencing degree of species was expressed by the length of the bar in the histogram; (**b**) Cladogram. The circle radiated inside out, demonstrated the classification—from the kingdom to species. Each small circle at different classification represents a taxon and the diameter of the circle is proportional to the relative abundance. The species not with significant differences are colored by yellow, and biomarkers are colored by different groups. Red and green dots represent the core bacterial populations in the respective group. Abbreviation: LDA, linear discriminant analysis.

To explore the bacterial taxa that may be responsible for the observed differences in community structure, a LEfSe was performed on the cecal community. There were 31 biomarkers with statistical differences in the linear discriminant analysis distribution histogram (Figure 8a). Among these, 18 biomarkers were enriched in CON, including B*acteroidales*, *Bacteroidota*, *Bacteroidia*, *Bacteroides_Barnesiae*, *Bacterium* et al. *Clostridia*, *Succinvibrionaceae*, *Aeromonadales*, *CHKCI001*, *Faecalibacterium* et al. were 13 biomarkers with the highest average richness corresponding to the CCHMA group. Also, the average relative abundance of each class detected in cecum samples was compared between two groups of geese (Supplementary Figures S1–S3).

The evolutionary branch graph indicated that the important microbial groups of CON are *Bacilli*. *Archaea* radiating outward to *Euryarchaeota*, *Methanobacteria*, *Methanobacteriales*, *Methanobacteriaceae*, *Methanobrevibacter*. *Bacteria* radiating outward to *Bacteroidota*, *Bacteroidia*, *Bacteroidales*. *Patescibacteria* radiating outward to *Saccharimonasia*, *Saccharimonadales*. The important microbial groups of the CCHMA group are *Clostridia*. *Eubacteriaceae* radiating outward to *Eubacterium*. *Peptostreptococcales_Tissierellales* radiating outward to *Peptostreptococcaceae*, *Romboutsia*. *Aeromonadales* radiating outward to *Succinivibrionaceae*.

#### 3.6.5. Associations of the Cecum Microbiota Composition with Growth Performance

**Figure 9:**
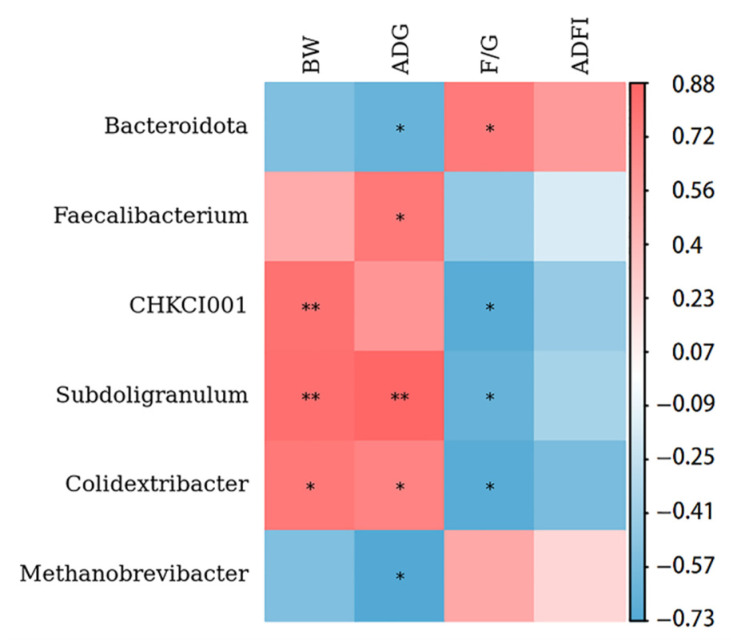
Heatmap of the spearman’s correlation coefficient between the cecal microbiota composition and growth performance indexes of geese. The red and blue color represents a positive and negative correlation, respectively. * *p* < 0.05, ** *p* < 0.01.

The relationships between the cecal microbiota composition and growth performance in geese were investigated with Spearman’s rank correlation coefficient analysis (Figure 9). The results showed that *Faecalibacterium*, *Colidextribacter* and *Subdoligranulum* were positively associated with the ADG, while *Bacteroidetes* and *Methanobrevibacter* were negatively associated with ADG (*p* < 0.05). The *CHKCI001*, *Colidextribacter*, and *Subdoligranulum* were meaningly positively correlated with the BW, but significantly negatively correlated with the F/G (*p* < 0.05). Finally, *Bacteroidetes* had significant positive correlations with F/G (*p* < 0.0*5*).

## 4. Discussion

The demand for geese as a source of meat for human consumption has increased in recent years. The main goal of poultry production is to obtain higher muscle yield. Antibiotics have been added to animal feed at sub-therapeutic doses to increase production performance and feed conversion efficiency, and to prevent infections for more than 60 years [24]. However, in recent years, the development of antibiotic resistance and antibiotic residues in food have become serious problems [25]; therefore, antibiotics are gradually banned as growth promoters in animal husbandry [26]. Moreover, the addition of antibiotics has been reported may have a long-term negative effect on the health of the host [27,28]. Hence, the discovery of alternatives to antibiotics that can improve animal production performance has important implications for food security and livestock. There is increasing evidence that CCHMA as feed additives can improve broiler production performance by regulating the gut microbiota composition [29,30], suggesting that the microbiota of gut plays an important role in host health. However, to our knowledge, no study to date has conducted the use of CCHMA in Zi geese. Different animals have different gut microbial compositions; conversely, different gut microbial compositions contribute to different production performances. Therefore, the effects of CCHMA on the growth performance, slaughter performance, serum biochemical indicators, intestinal morphology and cecal microbiota diversity of Zi goose were evaluated in this study.

Hesperidin in *Chenopodium* can accelerate the peristalsis of the gastrointestinal tract by increasing gastrin content and decreasing acetylcholine, motilin and vasoactive intestinal peptide levels [31], thus, promoting the digestion and absorption of feed in Zi goose. In the first feeding experiment, geese fed CCHMA at 3 g/kg had the highest BW and ADG. Through the second feeding experiment, it was found that the addition of 3 g/kg CCHMA improved BW, ADG and decreased F/G, but did not affect ADFI. This was in line with the research findings of Hundal et al. [32], using the herbs as feed additive, which improves the feed conversion efficiency and the health of livestock. And it was suggested that the improved the growth performance observed in this study was primarily the result of increased feed efficiency rather than increased feed consumption.

Slaughter performance is an important indicator to evaluate carcass quality and carcass yield of meat animals and poultry [33]. In this study, we found that the addition of 3 g/kg of a CCHMA to the Zi goose diet significantly increased the dressed yield of Zi goose, which was consistent with the results of Zi goose growth performance. The level of abdominal fat yield directly affects the taste and flavor of the meat. However, the consumption of fat from geese is usually not recommended by nutritionists because of excessive consumption of animal fats is not good for human health, although its high proportion of saturated fatty acids compared to other poultry [34]. In this study, the abdominal fat yield of Zi goose was reduced by adding 3 g/kg of a CCHMA into the Zi goose diet, which was consistent with the result of Kiramang et al. [35]. It was speculated that it may be because of the role of angelica, which has been proved to act as a nutritional supplement to improve lipid metabolism in diabetes due to high fat [36,37]. In addition, the addition of 3 g/kg of CCHMA to the Zi goose diet also had a tendency to improve the breast muscle yield of Zi goose.

The content of AST and ALT in the blood is often an indicator of liver function, which reflecting the permeability of the hepatocyte membrane [38,39]. The diet with CCHMA significantly reduced the AST and ALT levels in the serum of Zi goose. This is similar to the findings of Ran et al. [40] and Zhang et al. [41]. The hepatoprotective effect of CCHMA is mainly due to the action of antioxidant and anti-inflammatory activities of herbs [42].

The intestine is an important barrier against pathogens. Similar to broilers, the ileum of Zi goose is the main site of nutrient digestion and absorption [43]. Longer villus height reflects higher nutrient uptake capacity. Increasing the height of the ileal villus height of Zi goose would be beneficial in improving the rate of nutrient absorption [44]. In this study, 3 g/kg of CCHMA significantly increased the height of ileal villus height in Zi goose. This was due to the antibacterial properties of herbs, the active ingredients in herbs such as alkaloids, flavonoids, and essential oils, thus improving digestibility [45]. The interaction between herbs and the intestine was discussed by An et al. [46], who mentioned that herbs can convert large molecules in the intestine into small molecules, which will facilitate the pharmacological effects of herbs and improve the absorption and utilization of nutrients. In addition to this, *Aractylodes* as a polysaccharide called RAMPtp, which promotes the proliferation and survival of intestinal epithelial cells when stimulated by sodium dextran sulfate [47]. Inulin-type fructose in radix et rhizome ginseng also exhibited anti-inflammatory effects under the same stimulus [48]. This result indicates that CCHMA has a good protective and functional promoting effect on the intestinal tract of Zi goose.

The cecum of goose contains a complex ecosystem comprising of a highly diverse microbiome. Alpha diversity is usually used to estimate microbial community diversity in individual samples, whereas beta diversity generally evaluates the differences in the species complexity of samples at group levels. In the current study, no significant difference in the alpha diversity of the cecal microbiota were observed between the CON and the CCHMA group. Nevertheless, beta diversity revealed that the cecum microbiota composition was significantly different between CON and the CCHMA group, suggesting distinct differences in gut development and key phylotypes of the cecal microbiota between CON and the CCHMA group. Cecal digestion mechanism in geese is similar to that of a rumen; for example, the cecal microbiota of goose can digest cellulose to produce short-chain fatty acids (SCFAs), which are taken up by intestinal epithelial cells and serve as additional energy for the host [49]. Moreover, an increased ratio of *Firmicutes* to *Bacteroidetes* markedly affects energy gain in dairy cows [50]. *Lachnospiraceae*, *Ruminococcaceae*, and *Colidextribacter* are mainly SCFAs producing bacteria [29,30,51,52]. Here, higher abundances of *Firmicutes* to *Bacteroidetes* was found, *Romboutsia* (phylum *Firmicutes*), *CHKCI001* (family *Lachnospiraceae*), *Faecalibacterium* (family *Ruminococcaceae*), *Colidextribacter* and lower abundances of *Bacteroidetes* in the cecal contents of geese in the CCHMA group than in those of in CON group. In addition, the ileum villus height/crypt depth of geese was also higher in the CCHMA group than in the CON group. A higher ileum villus height/crypt depth, indicating a greater gut nutrient absorption capacity [53]. These results advised that the geese in the CCHMA group have outstanding nutrient absorption abilities than the geese in the CON group.

Therefore, compared with the CON group, geese in the CCHMA group had better growth performance (higher BW, ADG, and lower F/G ratio), slaughter performance (higher dressed yield) and lower serum biochemical indicators (AST and ALT). Moreover, the relative abundances of *Euryarchaeota* and *Methanobrevibacter* (phylum *Euryarchaeota*) were significantly higher in the geese of the CON group than that of the CCHMA group. *Methanobrevibacter* is the main methanogen, and it is associated with a loss of gross energy intake [54]. This may be the reason why the ileum villus height/crypt depth was lower in the CON group geese than in the CCHMA group geese. Subsequently, the study explored the relationship between the cecal microbiota composition and the growth performance in geese by Spearman’s rank correlation coefficient analysis and found that *CHKCI001*, *Faecalibacterium*, *Colidextribacter*, and *Subdoligranulum* were positively associated with the growth performance. These genera can provide additional energy to the host, as mentioned earlier. On the other hand, *Bacteroidetes* and *Methanobrevibacter* were negatively correlated with ADG. These results suggested that the cecal microbiota was closely related to nutrient absorption and plays a key role in the growth performance of geese.

To date, information is limited regarding the specific physiological proportions of these bacterial groups. Further evaluation of their effects on gut microbiota of birds is needed. And further studies are required to determine the mod of action of CCHMA.

## 5. Conclusions

This study demonstrated that adding 3 g/kg of CCHMA to the diet can improve the important parameters of growth performance, slaughter performance and serum biochemical indexes of Zi goose. Also, 3 g/kg of CCHMA improved ileal villus height and increased beneficial flora content of cecum. These results provide valuable information for the development of CCHMA. Therefore, adding 3 g/kg CCHMA to goose’s diet is a good growth promoter.

## Figures and Tables

**Table 1:** Ingredients and nutrient levels of the experimental diets.

Ingredients	Content (%)
Corn	50.80
Soybean meal	18.95
Corn protein meal	4.15
Wheat bran	7.20
Rice bran	10.90
Limestone	1.70
CaHPO4	0.80
NaCl	0.35
Met	0.15
Lys	0.25
Choline	0.15
^1^ Premix	2.00
^2^ Nutrient levels	
ME (MJ/kg)	10.85
CP (g/kg)	17.16
Crude fiber (g/kg)	5.88
Crude ash (g/kg)	5.08
Ca (g/kg)	0.80
TP (%)	0.37
Met (%)	0.35
Lys (%)	0.65

^1^ Supplied per kilogram of premix: vitamin A, 15,000 IU; vitamin D_3_, 2500 IU; vitamin E, 20 mg; vitamin K_3_, 2 mg; vitamin B_1_, 1.6 mg; vitamin B_2_, 6.5 mg; vitamin B_6_, 3.25 mg; vitamin B_12_, 0.15 mg; niacin, 28 mg; pantothenic acid, 8 mg; folic acid, 0.8 mg; biotin, 75 μg; Fe, 50 mg; Cu, 4 mg; Zn, 50 mg; Mn, 80 mg; I, 1.3 mg; Se, 18 mg. ^2^ Nutrient levels are calculated values.

## Data Availability

The dataset generated and/or analyzed during the current study is available from the corresponding author on reasonable request.

## Supplementary Materials

The following supporting information can be downloaded at: https://www.mdpi.com/article/10.3390/ani12212942/s1, Figure S1: histogram of the abundance of samples with different bacterial groups (1); Figure S2: histogram of the abundance of samples with different bacterial groups (2); Figure S3: histogram of the abundance of samples with different bacterial groups (3); Table S1: sequencing depth of caecal flora of Zi geese in control and experimental group.
