# Supplementary material for: Effects of Compound Chinese Herbal Medicine Additive on Growth Performance and Gut Microbiota Diversity of Zi Goose
Source: Animals (Basel). 2022 Oct 26;12(21):2942. doi: 10.3390/ani12212942 (PMC9655946; doi:10.3390/ani12212942)
Supplement: Supplementary file 1 [file animals-12-02942-s001.zip › Table S1.pdf]

Supplementary Table S1. Sequencing depth of caecal flora of Zi geese in control and experimental group

Items	Raw PE	Clean Tags	Effective Tags	Q20	Q30
CON1	80490	78875	64747	97.66	92.83
CON2	92597	90753	69956	97.67	92.89
CON3	87755	86498	65819	97.66	92.80
CON4	83998	82255	64425	97.69	92.81
CON5	82005	80877	64407	97.70	92.87
CCHMA1	89122	86864	67383	97.73	92.86
CCHMA2	80686	79010	62887	97.81	93.11
CCHMA3	88782	87443	69751	97.65	92.89
CCHMA4	88607	87045	69586	97.72	92.91
CCHMA5	96284	95138	70977	97.90	93.25

Abbreviation: Raw PE, the original PE reads; Clean Tags, sequences after filtering low quality and short length; Effective Tags, the final Tag sequence used for subsequent analysis after filtering chimeras; Q20, the percentage of bases with base quality value greater than 20 (sequencing error rate less than 1%) in Effective Tags; Q30, the percentage of bases with base quality value greater than 30 (sequencing error rate less than 1%) in Effective Tags.
