# Supplementary figures and images for: Effects of Compound Chinese Herbal Medicine Additive on Growth Performance and Gut Microbiota Diversity of Zi Goose
Source: Animals (Basel). 2022 Oct 26;12(21):2942. doi: 10.3390/ani12212942 (PMC9655946; doi:10.3390/ani12212942)

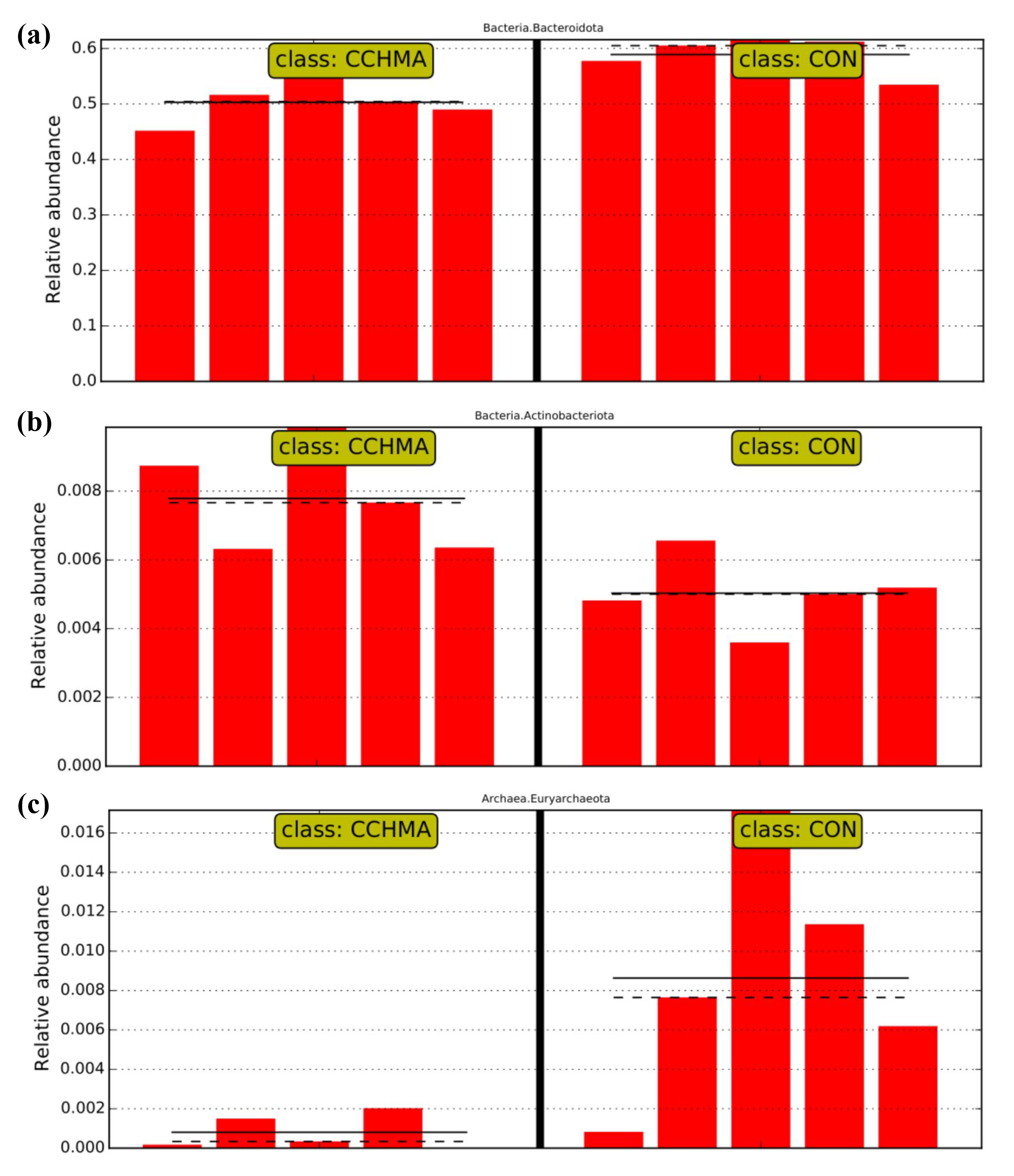

Supplement: Supplementary file 1 [file animals-12-02942-s001.zip › Figure S1.tif]

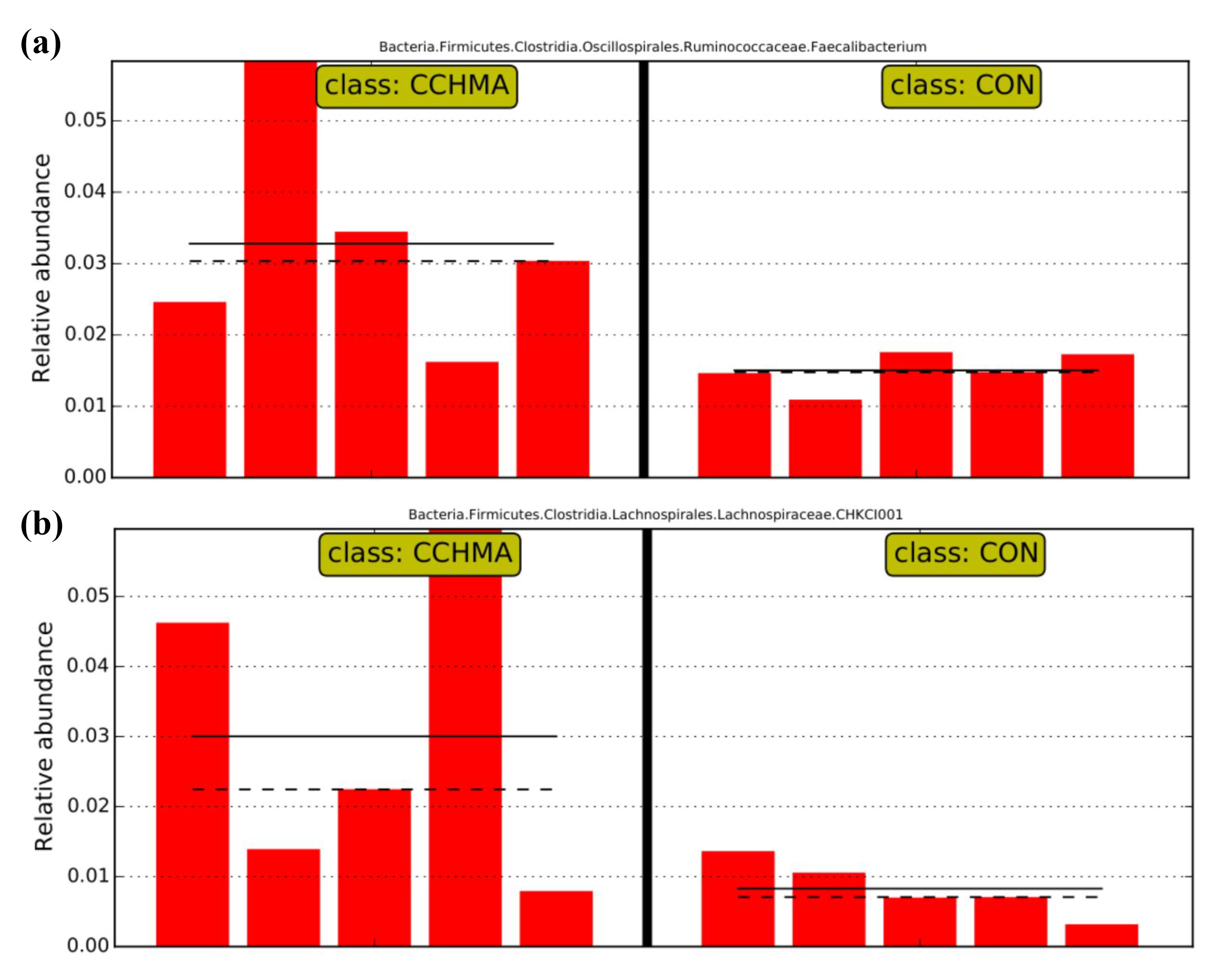

Supplement: Supplementary file 1 [file animals-12-02942-s001.zip › Figure S2.tif]

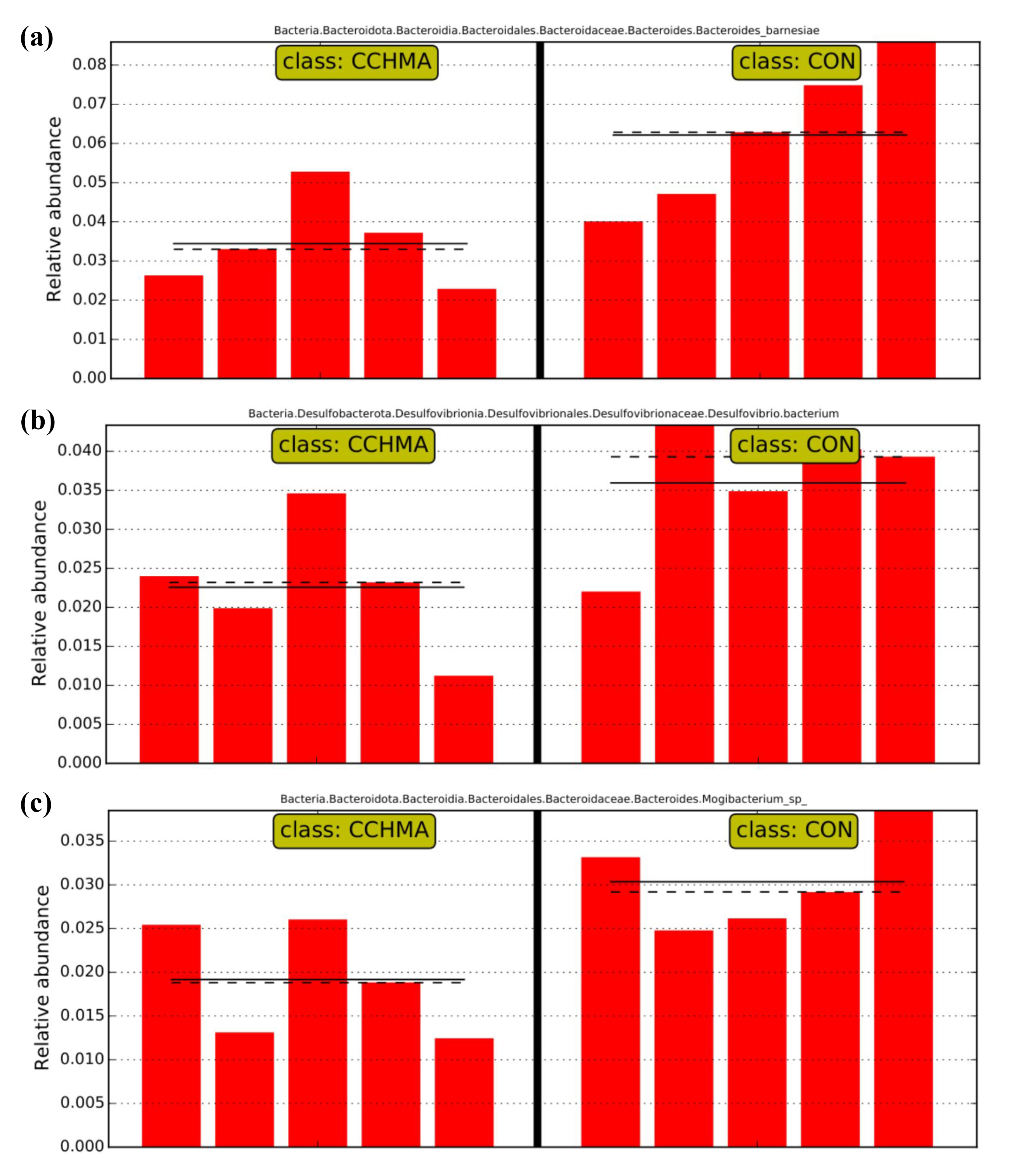

Supplement: Supplementary file 1 [file animals-12-02942-s001.zip › Figure S3.tif]
